# Patchy Core/Shell, Magnetite/Silver Nanoparticles via Green and Facile Synthesis: Routes to Assure Biocompatibility

**DOI:** 10.3390/nano10091857

**Published:** 2020-09-17

**Authors:** Carlos M. Ramírez-Acosta, Javier Cifuentes, Juan C. Cruz, Luis H. Reyes

**Affiliations:** 1 Grupo de Diseño de Productos y Procesos (GDPP), Department of Chemical and Food Engineering, Universidad de los Andes, Bogotá 111711, Colombia; cm.ramirez10@uniandes.edu.co; 2 Department of Biomedical Engineering, Universidad de los Andes, Bogotá 111711, Colombia; jf.cifuentes10@uniandes.edu.co; 3 School of Chemical Engineering and Advanced Materials, The University of Adelaide, South Australia 5005, Australia

**Keywords:** patchy nanoparticles, core/shell, green synthesis, biocompatibility

## Abstract

Nanomedicine is entering a high maturity stage and is ready to reach full translation into the clinical practice. This is because of the ample spectrum of applications enabled by a large arsenal of nanostructured materials. In particular, bimetallic patchy core/shell nanoparticles offer tunable surfaces that allow multifunctional responses. Despite their attractiveness, major challenges regarding the environmental impact and biocompatibility of the obtained materials are yet to be solved. Here, we developed a green synthesis scheme to prepare highly biocompatible patchy core/shell magnetite/silver nanoparticles for biological and biomedical applications. The magnetite core was synthesized by the co-precipitation of ferric chloride and ferrous chloride in the presence of NaOH. This was followed by the patchy silver shell’s growth by a green synthesis approach based on natural honey as a reducing agent. A purification process allowed selecting the target patchy nanoparticles and removing excess toxic reagents from the synthesis very efficiently. The obtained patchy magnetite/silver nanoparticles were characterized by UV-Vis spectrophotometry, dynamic light scattering (DLS), thermogravimetric analysis (TGA), Fourier transform infrared spectroscopy (FTIR), scanning electron microscope equipped with energy-dispersive spectroscopy (SEM + EDS), and transmission electron microscopy (TEM). The morphology, patchiness level, and size of the nanoparticles were determined via SEM and TEM. In addition, the spectrophotometric characterization confirmed the presence of the patchy silver coating on the surface of the magnetite core. The nanoparticles show high biocompatibility, as evidenced by low cytotoxicity, hemolytic effect, and platelet aggregation tendency. Our study also provides details for the conjugation of multiples chemistries on the surface of the patchy bimetallic nanoparticles, which might be useful for emerging applications in nanomedicine, where high biocompatibility is of the utmost importance.

## 1. Introduction

Nanomedicine is currently approaching the maturity level required to translate into the clinical practice fully. This has been attributed to our increasing ability to synthesize and modify objects at the nanoscale with relatively high precision [1,2,3]. As a result, numerous applications ranging from biosensors to drug and gene delivery now hold much promise to impact patients’ health positively [1,2]. One example of complex nanostructured objects with potential nanomedicine applications is bimetallic nanoparticles, combining attractive features of two different metallic elements arranged in unique supramolecular structures [4,5,6]. Examples include bimetallic nanoparticles combining nickel and cobalt, and silver and platinum [7,8]. In the first case, the nanostructure was formed by the microwave irradiation synthesis method and exploited the magnetism of nickel and the solid and liquid solubility of cobalt in a core/shell arrangement [7]. In contrast, in the second case, the main properties were antioxidant and reactive oxygen species, and the formed silver–platinum structure was obtained by a green synthesis [8]. Finally, another patchy core/shell system for nanomedicine applications is magnetite/titanium oxide, which has proven useful in the highly selective enrichment of phosphopeptides [9].

Patchy core/shell nanoparticles have attracted significant attention, mainly due to their tunable surface and the properties of both the core and metallic shells [4,10]. Features such as magnetism and chemical compatibility can be combined relatively straightforwardly, thereby making them of great interest in nanomedicine applications such as antibacterial systems, imaging agents, and drug delivery carriers [4,10,11]. Moreover, they can be functionalized with different macromolecules, including polymers, peptides, antibodies, and even nucleic acids [12,13]. For instance, the obtained nanoconjugates have found applications in the ultrasensitive detection of biological species or the targeted delivery of nucleic acids at the subcellular level [14,15]. Most patchy core/shell nanoparticles’ synthesis methods mainly rely on bottom–up approaches where chemical precursors can self-assemble into the desired arrangement in multi-stage processes with controlled conditions [16,17,18,19]. Materials obtained by this method generally exhibit soft units with flexible bonding patterns and sizes in the range of 1 to 100 nm. Top–down routes are not as popular mainly because the resulting materials exhibit pre-defined and fixed patchiness, and they rely on sophisticated equipment for manufacturing and processing [17,19,20]. Some of the bottom–up synthesis schemes include electrochemical, sonochemical, and thermal decomposition, which require high energy levels and controlled conditions such as inert atmospheres [21,22,23]. For example, the electrochemical synthesis to manufacture silver shells on gold nanoparticles and the sonochemical route to synthesize magnetite core with a deposition of superficial silica [21,22,23].

Compared to conventional synthesis schemes, the greener ones mainly encompass a growth media or a natural organic compound [24,25]. Consequently, during the shell or coating formation, the crude extract’s quality assurance plays a major role, as it defines the functional groups interacting, which ultimately controls the level of patchiness and the homogeneity of the final nanomaterial. Several synthesis methods produce the patchy core/shell made of metal/metal systems, including ligand selection, direct and self-assembly, physical or chemical absorption, and seeded growth [11,25]. Due to its flexibility, versatility, and relatively low costs, seeded-growth schemes have been extensively implemented [19,26]. However, the need for environmentally friendly and readily accessible synthesis methods led to greener versions of previously mentioned synthesis schemes [11,24,25]. As a result, novel green synthesis routes have been successfully developed to manufacture core/shell bimetallic nanoparticles, such as gold/silver and magnetite/silver [24,27,28]. Moreover, most studies report methodologies involving anaerobic conditions or drying out the final nanomaterial to remove residues of the natural extract [23,25]. Thus, the purification process represents one of the main challenges regarding the full potential of extract-based green syntheses.

Therefore, this study is dedicated to developing a facile method to synthesize patchy core/shell magnetite/silver nanoparticles, starting for magnetite cores as seeds and followed by a green synthesis of the patchy silver shell [29,30]. In addition, the patchy core/shell system proposed here focuses on a tunable surface as an advantage for biomedical technologies where multiple chemistries and functional groups might be required. The obtained materials exhibit properties similar to those described by Haiyang et al. in their review about core/shell nanoparticles [31]. We also described a purification process to assure that potentially cytotoxic chemical species are entirely removed from the nanoparticle suspensions. Moreover, the nanoparticles were characterized via electron microscopy and thermal and spectroscopic techniques. Our study also provides details for the conjugation of multiples chemistries on the surface of the patchy bimetallic nanoparticles, which might be useful for emerging applications in nanomedicine, where high biocompatibility is of the utmost importance.

## 2. Materials and Methods

### 2.1. Synthesis and Characterization of the Magnetite Core

**Figure 1:**
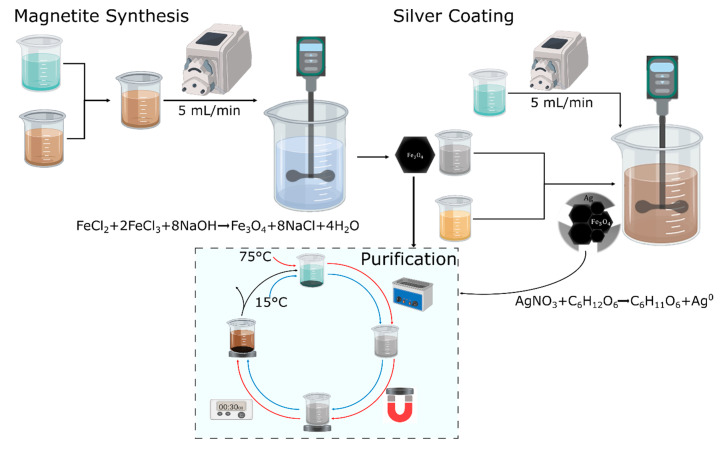
Schematic for the green synthesis of patchy core/shell, magnetite/silver nanoparticles. First, magnetite was synthesized using the co-precipitation method (the reaction scheme taking place is also shown). Second, the patchy silver coating was deposited by reducing silver nitrate in a honey solution (reducing agent) with the glucose present (reaction shown below). Finally, the dotted frame process represents the cleaning or purification steps with either hot (75 °C) or cold water (15 °C).

The synthesis was conducted via the co-precipitation method using iron salts as a precursor. Iron (II) chloride and iron (III) chloride were purchased from J. T. Baker (USA) and Merck (Kenilworth, NJ, USA), respectively. Briefly, a 2 Fe(II):1 Fe(III) molar ratio solution was added dropwise to a sodium hydroxide solution 5 M at 90 °C (PanReac AppliChem, Darmstadt, Germany) at a rate of 5 mL/min, as shown in Figure 1: Magnetite synthesis. Mechanical agitation was kept at 400 RPM with a Rushton impeller in a 600 mL beaker, while the iron solutions were added. An ultrasonic bath was used to resuspend the synthesized nanoparticles. The excess reagents left from an incomplete reaction were removed, in the first place, by placing a neodymium magnet below a precipitation beaker, thereby accelerating the magnetic nanoparticles precipitation. Secondly, the supernatant was removed and discarded, and finally, water at approximately 75 °C was added, followed by resuspension in an ultrasonic bath. The procedure was repeated several times until the supernatant was colorless (Figure 1: Purification). Finally, the washed nanoparticles were resuspended in Milli-Q water. Bare magnetite nanoparticles were imaged via scanning electron microscope (SEM, TESCAN LYRA3 FIB-SEM, Czech Republic) and subsequently characterized by UV-Vis spectrophotometry (Thermo Scientific GENESYS, Waltham, MA, USA). Dynamic light scattering (DLS) was used to determine the hydrodynamic diameter (Zeta-Sizer Nano-ZS, Malvern Panalytical, Malvern, UK). The UV-Vis absorbance spectra were collected for a suspension of the nanoparticles in Type-I water (0.1 mg/mL) from 200 to 1000 cm^−1^.

### 2.2. Silver Shell Formation

A redox reaction, starting with 1 mM silver nitrate (Merck, Kenilworth, NJ, USA) and 20% (*w*/*v*) aqueous honey solution from a local farmer’s market (as a reducing agent), was used to create the silver shell via the developed green deposition scheme. The procedure consists in dissolving 5 mL of magnetite (1 mg/mL) in the honey solution followed by pumping the silver nitrate into the suspension at 5 mL/min. Magnetite structural integrity can be compromised when pH descends below 4, and for that reason, it was maintained above 4 with the aid of NaOH. Once the silver nitrate solution was added, the pH was adjusted to 8 using a NaOH solution (1M). The experimental setup for the synthesis is shown in Figure 1: Silver Coating. Once the silver coating was completed, the obtained nanoparticles were suspended in an organic compounds’ solution remaining from the synthesis. Commercially available (from a local producer) honey’s organic compounds include acetic acid, citric acid, vitamin B, and glucose. The obtained nanomaterials were most likely a mixture of uncoated magnetite, free silver nanoparticles, and the patchy magnetite/silver nanoparticles.

### 2.3. Magnetite/Silver Purification and Physical Characterization

In order to purify the patchy magnetite/silver nanoparticles, free silver nanoparticles were separated from magnetite and magnetite/silver via magnetic precipitation with the aid of a neodymium magnet by following the cleaning steps described above (Figure 1: Purification). Then, the remaining bare magnetite was precipitated using hydrochloric acid (1 mM) until the pH reached 3. Under such conditions, magnetite nanoparticles change their crystalline structure and tend to form an iron salt, which lacks magnetic properties. In contrast, under the same conditions, the patchy magnetite/silver nanoparticles preserve their magnetic responsiveness and can be separated magnetically, as discussed above in the magnetite synthesis section (Figure 1: Purification). The obtained magnetite/silver nanoparticles were first characterized by determining their hydrodynamic radii via DLS. In addition, the presence of the silver shell was verified by collecting the absorbance spectrum from 200 to 1000 cm^−1^ and comparing it with that of magnetite, which is aided by a UV-Vis spectrophotometer. The nanoparticles of magnetite and magnetite/silver were imaged, and their atomic composition was determined using a scanning electron microscope (SEM + EDS, TESCAN LYRA3 FIB-SEM, Czech Republic), which was equipped with energy-dispersive spectroscopy. A transmission electron microscope (TEM) Tecnai F30 (FEI Company, Fremont, CA, USA), at a resolution of 134 eV and reference energy of 5.9 keV, was used to analyze the magnetite/silver coverage.

### 2.4. Magnetite/Silver Surface Chemistry

In order to evaluate the chemical reactivity of both surfaces, magnetite and silver, two different molecules were conjugated: first, an organosilane on the uncovered magnetite, and second, a cationic polymer on the silver shell. The organosilane (3-aminopropyl) triethoxysilane (APTES, 98%, Sigma-Aldrich, St. Louis, MO, USA), a molecule commonly used to anchor on the surface of the magnetite, was conjugated by the addition of 200 μL of APTES to suspensions of magnetite and magnetite/silver nanoparticles. The conjugation proceeded with sonication at 20 kHz for 30 min, using a sonic tip. To finalize the conjugation, the APTES that was not covalently attached to the nanomaterial surface was removed by following the purification process mentioned earlier, where water was added at approximately 75 °C, followed by magnetic precipitation (Figure 1: Purification). On the silver shell surface, the cationic polymer poly(2-dimethylamino)ethyl methacrylate) methyl chloride quaternary salt (pDMAEMA) purchased from Sigma-Aldrich (USA) was conjugated. First, the polymer was dissolved in an aqueous solution (10 mg/mL). The purified magnetite/silver nanoparticles were stirred at 500 RPM and 50 °C for 1 h. With the chlorine groups left from the purification process, a Hofmann elimination reaction was carried out to conjugate the pDMAEMA. The conjugation of APTES and pDMAEMA was verified using Fourier transform infrared spectroscopy (FTIR) in a Bruker Alpha II FTIR Eco-ATR instrument (Bruker, Billerica, MA, USA) by collecting the spectra from 900 to 4000 cm^−1^ and comparing them directly to the patchy bimetallic nanoparticles. Before collecting the data, once again, the nanoparticles were purified using magnetic precipitation (Figure 1: Purification). Thermogravimetric analyses (TGA), in a simultaneous TGA/DSC instrument (TA Instruments, New Castle, DE, USA), were conducted to estimate the pDMAEMA and APTES conjugation efficiencies, implementing a linear temperature ramp at a rate of 10 °C/min from 25 to 600 °C under an inert atmosphere.

### 2.5. Biocompatibility

The hemolytic activity of magnetite and magnetite/silver bimetallic nanoparticles was tested on erythrocytes isolated from a healthy human donor’s freshly drawn blood. The erythrocytes were collected by centrifuging the blood at 1800 RPM for 5 min, discarding the plasma, and finally washing them five times with NaCl solution (0.9% (*w*/*v*)) and once with PBS (Phosphate Buffered Saline) (1X). An erythrocytes stock was prepared by adding 1 mL of isolated erythrocytes (4.3 × 10^6^ erythrocytes/μL) in 9 mL of PBS (1X). Serial dilutions of the magnetite and the patchy magnetite/silver nanoparticles (200 to 12.5 μg/mL) were prepared by mixing initial stocks with PBS (1X). Triton X-100 (1% (*v*/*v*)) and PBS (1X) were used as positive and negative controls, respectively. Erythrocytes (100 μL) were seeded in a 96-well microplate, exposed to each treatment (100 μL), incubated at 37 °C, 5% CO_2_ for one hour, and centrifuged at 1800 RPM for 5 min. Then, 100 μL of each supernatant was read at 450 nm in a microplate reader. The hemolysis percentage was calculated using Equation (1).

(1) $$Hemolysis\;percentage=\frac{Abs_{450}\left(sample\right)-Abs_{450}\left(negative\;control\right)}{Abs_{450}\left(positive\;control\right)-Abs_{450}\left(negative\;control\right)}\times 100$$

The platelet aggregation tendency of magnetite and patchy magnetite/silver nanoparticles was tested on platelets isolated from a healthy human donor’s freshly drawn blood. A vacutainer tube was used to collect the blood sample containing sodium citrate as an anticoagulant. A platelet-rich plasma (PRP) was obtained by centrifuging the sample at 1000 RPM for 15 min at room temperature (25 °C). Serial dilutions of the magnetite and magnetite/silver nanoparticles (200 to 12.5 μg/mL) were prepared by mixing concentrated stocks with PBS (1X). As a positive control, thrombin (6 U) was used, and PBS (1X) served as a negative reference. Then, 50 μL of PRP was mixed with 50 μL of the different samples in a 96-well microplate and incubated at 37 °C, 5% CO_2_ for 5 min. Finally, a sample of 50 μL of each supernatant was extracted, and absorbance was read at 620 nm in a microplate reader.

The quantification of the lactate dehydrogenase enzyme (LDH), using a commercially available Cytotoxicity Detection Kit (LDH) (Roche, Basel, Switzerland), was used to determine the cytotoxicity of purified patchy magnetite/silver nanoparticles. The cytotoxicity was tested by exposing the samples to Vero cells (ATCC^®^ CCL-81) at a cell density of 100,000 cells/mL. Serial dilutions (i.e., 200–12.5 μg/mL) were prepared by mixing concentrated stocks with DMEM (Dulbecco’s Modified Eagle’s medium) media. Triton X-100 (1% (*v*/*v*)) was used as positive control, and DMEM media was used as the negative one. Then, 100 μL of the cell stock (DMEM, supplemented with 10% (*v*/*v*) Fetal Bovine Serum-FBS) were deposited in 96-well microplates (10,000 cells/well) and incubated at 37 °C, 5% CO_2_ for 24 h. After incubation, culture media was removed, and cells were washed with PBS (1X). Later, PBS was removed, and 100 μL of the different samples were added and incubated at 37 °C, 5% CO_2_ for 24 and 48 h. Then, 50 μL of the supernatants were transferred to 96-well microplates along with 50 μL of the reaction mixture (Cytotoxicity Detection Kit (LDH), Roche, Basel, Switzerland) and left to react under orbital stirring (50 RPM), room temperature, and complete darkness for 15 min. Absorbance was finally read at 490 nm in a microplate reader. The cytotoxicity was compared to that reported for magnetite in previous studies.

## 3. Results and Discussion

### 3.1. Morphology and Elemental Composition

**Figure 2:**
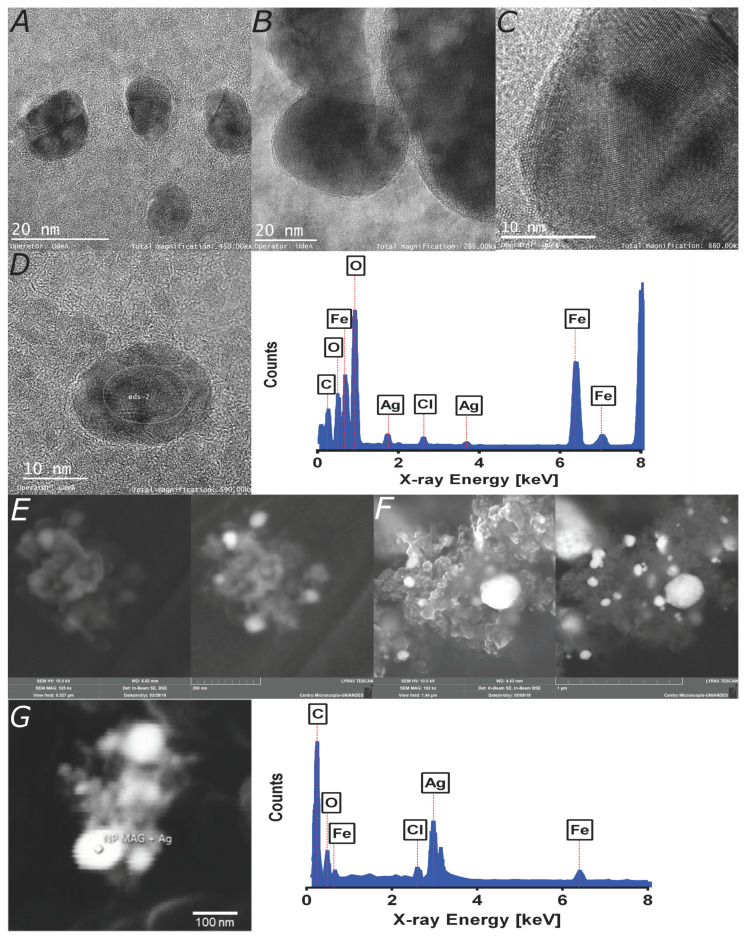
**Microscopy imaging and elemental characterization of TEM images.** (**A**) Magnetite/silver nanoparticles are exhibiting an average diameter of 11.7 nm. (**B**) Magnetite clusters covered with a thin silver layer. (**C**) Patchy magnetite/silver nanoparticles as confirmed by the interplanar distances of the corresponding crystalline structures. (**D**) Elemental composition of the marked region, which confirms the presence of iron, silver, and chlorine. SEM images: (**E**) A core magnetite nanoparticles cluster synthesized by co-precipitation (scale bar corresponds to 200 nm). (**F**) Patchy magnetite/silver nanoparticles show a definite change in morphology. White spots are most likely related to the silver coverage (scale bar corresponds to 1 μm). (**G**) The marked region’s elemental composition verifies the presence of oxygen, iron, and silver, which are all related to the patchy bimetallic nanoparticles and chlorine remaining from the purification process. The carbon content is due to the graphite adhesive tape (scale bar corresponds to 100 nm).

In order to investigate the size, size distribution, clustering, morphology, and silver shell coverage, TEM imaging was implemented. Figure 2A–C show the TEM images of patchy magnetite/silver nanoparticles. The individual patchy magnetite/silver particles’ average size approaches 11.7 nm, which agrees well with sizes reported elsewhere for similar coatings [32]. Arguably, the magnetite core’s silver coating is unevenly distributed on the surface and is formed around clusters of magnetite nanoparticles, as it can be evidence from Figure 2B–C. These results agree well with those of Garza-Navarro et al., who also found a heterogeneous silver coverage [28]. In addition, the micrographs suggest that the coating varies from cluster to cluster.

Additionally, an SEM magnification was conducted (Figure 2E–F). The beam was focused on a cluster of nanoparticles, which shows the morphology of an agglomerate. There is an evident difference in size between the TEM and SEM results due to the magnification of each technique. While the TEM verifies the coating patchiness, SEM shows that there are regions with a major presence of silver, thereby suggesting marked differences in the patchiness between individual nanoparticles and clusters.

Besides inspecting the morphology, we conducted an Energy-dispersive X-ray spectroscopy (EDX) analysis of the nanoparticles (Figure 2D). The analyses confirmed the presence of iron and oxygen related to the magnetite core, silver, and chlorine from the shell, and possible HCl residues from bare magnetite nanoparticles’ purification. The chlorine atoms on silver nanoparticles or bimetallic nanoparticles surface provide reactive sites for the subsequent conjugation of different macromolecules. For instance, Hu et al. conjugated a nitrile to the chlorine atoms on silver nanoparticles to label the HeLa cells’ surface expressing a unique recombinant receptor [33]. The presence of silver and the chlorination extent were qualitatively assessed with the aid of the energy-dispersive spectroscopy (EDS) detector of the SEM instrument. The chlorine signal in Figure 2D–G corroborates chlorination’s success and, therefore, the suitability for further functionalization [34]. The results also suggest that the purification process proved successful at separating bare nanoparticles of silver. This process is mainly enabled by the magnetite/silver’s magnetic properties, which corroborated that the magnetism conferred by the magnetite core remained even after the silver coating. Additional studies are required to quantify the silver shell effect on magnetism.

### 3.2. Magnetite/Silver Characterization

**Figure 3:**
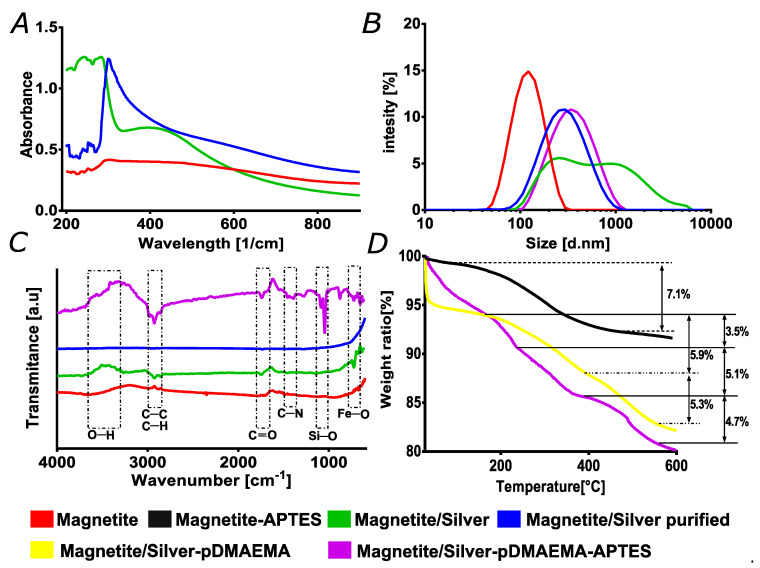
**Characterization and purification process** of magnetite/silver without purification (green). (**A**) UV-Visible spectra were collected to verify the silver coverage on magnetite nanoparticles, plus the effect of purification toward sample homogeneity. (**B**) Nanoparticles size distribution via dynamic light scattering. The data were useful to verify the homogeneity of the sample. (**C**) Fourier transform infrared spectroscopy (FTIR) spectra were used to evaluate the effectiveness of purification and cleaning of the nanoparticles and verify the presence of both chemistries on the magnetite/silver-pDMAEMA-APTES nanoparticles. Highlighted regions corresponding to pDMAEMA are C=O, C-C, and C-H bonds. Simultaneously, the silanization is corroborated by Si-O and C-N bonds, which are related to the conjugated pDMAEMA and APTES. The Fe-O bonds emerge from the magnetite core. (**D**) Thermogravimetric analyses (TGA) were also conducted to verify the effectiveness of conjugation for the magnetite-APTES, magnetite/silver-pDMAEMA, to magnetite/silver-pDMAEMA-APTES. APTES: (3-aminopropyl) triethoxysilane, pDMAEMA: poly(2-dimethylamino)ethyl methacrylate) methyl chloride quaternary salt.

Evidence provided by the UV-Vis data shown in Figure 3A supports the SEM and TEM results, thereby suggesting an incomplete or discontinuous coating of the silver shell. The silver shell presence was determined spectrophotometrically by collecting the absorbance in the 200–800 cm^−1^ range. Figure 3A shows the characteristic spectra of silver nanoparticles; the absorbance between 300 and 400 cm^−1^ increased compared to bare magnetite [22,32]. Still, the magnetite spectrum is visible in the range below 300 cm^−1^, indicating a discontinuous silver coverage [22,35]. Figure 3B presents the results of the hydrodynamic radii. Bare magnetite nanoparticles presented an average hydrodynamic diameter of 122 nm but increased to 295 nm after forming the silver shell. The corresponding polydispersity indexes (PI) increased from 22.6% to 43.5%, which was comparable with those of magnetite nanoparticles previously used to conjugate an antimicrobial peptide [36,37]. A fascinating finding was the importance of the purification process toward the sample homogeneity, as evidenced by the UV-Vis spectra and the DLS data before and after purification. In this regard, Figure 3A–B (green) show data for the synthesized materials before purification. In the UV-Vis case, the observed absorbance is most likely due to the silver plasmon, as reported for similar materials elsewhere [24,32]. The particle size distribution shows that before purification, the sample exhibited significant clustering with particles that even reached the micron size range. Similar distributions have been observed previously for materials with different metal components due to different surface charge values [22,38]. The FTIR data in Figure 3A confirmed the purification process’s effectiveness as peaks associated with organic compounds significantly decreased or even disappeared.

The nanomaterials capable of withholding different chemistries on their surfaces, such as antibodies, small interfering RNA (siRNA), or peptides, are of great interest, particularly in the medical field [39,40]. In our case, we are interested in the conjugation of translocating peptides and proteins for efficient cell penetration and endosomal escape. After synthesis, we expected that our patchy bimetallic nanoparticles exhibited the surface of both metals exposed for further conjugation. This was tested by conjugating pDMAEMA to silver, which is a polymer used for cell penetration and endosomal escape thanks to its pH-responsiveness. In this case, conjugation proceeded via Hoffman elimination and the chlorine chemistry [41,42]. As for the magnetite, the surface was evaluated by the conjugation of APTES, an organosilane molecule with an amine terminal that has been widely exploited for surface modification, as reported by Mohammed et al. in his review on magnetic nanoparticles in biomedical applications [40]. FTIR analyses were conducted to verify the successful conjugation by looking at the functional groups’ presence from the conjugated molecules on the surface of the nanoparticles. Figure 3C compares the spectra of magnetite, magnetite/silver before purification, purified magnetite/silver, and the purified nanoparticles after conjugation with the APTES and pDMAEMA. The C-C and C-H stretching vibrations in the magnetite/silver-pDMAEMA nanoparticles spectrum at around 3390 cm^−1^ are related to the pDMAEMA structure. This is also the case for the peaks at 2857 and 2929 cm^−1^, corresponding to C-C’s main chain and C-H stretching vibrations, respectively [43,44]. This result agrees well with similar characterizations for this polymer’s conjugation by Liu et al. [44].

Moreover, the stretching vibration at 1740 cm^−1^ corresponds to the carbonyl C=O group. At 1428 cm^−1^, the deformational stretching vibration of the -N(CH_3_)_2_ also belongs to the pDMAEMA [35,43]. Silanization, in this case, is the process by which the APTES binds covalently to the magnetite core of the patchy nanoparticles [45]. However, there exists a possibility for electrostatic interactions between the terminal amine group of APTES and the silver coating. We believe that, to a large extent, such interactions might be disrupted during the purification process by the thermal energy of hot water. Further studies are needed to estimate the relative fraction of adsorbed APTES compared with that covalently linked [46]. The peaks at 1065 and 950 cm^−1^ are for the Si-O-Si (due to silanization), and Fe-O bonds can be attributed to the magnetite core, respectively [30]. The silane chemistries on the nanomaterials’ surface are widely used in the medical field for several applications ranging from delivering nucleic acids or detecting biological markers [35,39]. As previously discussed, one can highlight the importance of the purification process by evaluating DLS and UV-Vis results. As shown in Figure 3, the contrast of the blue and green plots demonstrate that bare nanoparticles’ noise disappears in the UV-vis spectra, thereby displaying a well-defined peak when purified. The effectiveness of the process was corroborated by analyzing the extent of distribution of the hydrodynamic radii, where it is evident that the polydispersity of the sample decreases. Moreover, the FTIR analysis clearly shows the removal of organic compounds absorbed by the nanoparticles, as evidenced by a flat line representing the absence of major contaminants or impurities.

Thermogravimetric analyses were also conducted to estimate conjugation efficiencies (Figure 3D). With each sample’s respective weight, all nanoparticles’ samples exhibited an initial weight loss before 100 °C, which is most likely due to the moisture adsorbed. As calculated from the weight loss, bare magnetite was conjugated with APTES with an efficiency of around 7.1%. This result agrees well with previously reported studies [37]. Patchy magnetite/silver was conjugated, at first, with pDMAEMA, which showed a second weight loss of about 5.9%. This is probably related to excess reagents adsorbed by the nanoparticles and the polymer. The third weight loss started at around 400 °C and was 5.3% of the total weight, which was most likely due to the polymer, as shown in studies involving the thermogravimetric analysis of pristine pDMAEMA [35,47]. Finally, the conjugation of both molecules (i.e., pDMAEMA and APTES) on the patchy magnetite/silver nanoparticles showed that the second weight loss approached 8.6%, which can be attributed to the release of APTES and the adsorbed excess reagents on both the nanoparticles and the polymer, which was similar to that observed for the conjugation of the polymer. A final weight loss step above about 400 °C might be associated with the polymer pDMAEMA conjugated on the silver surface with 4.7% efficiency, which is comparable to previous reports [35,47]. Both FTIR and TGA confirmed the tunability of the surface of the patchy core/shell material synthesized, which was comparable to similar systems put forward previously in nanomedicine applications [31,48]. Thus, the patchy nanostructured material introduced here holds significant promise as an enabler of medical and biological applications. For instance, it is possible to conjugate individually or co-immobilize an ample range of biomolecules including DNA, RNA, antibodies, and recombinant proteins for applications ranging from sensing and diagnostics to highly targeted therapeutics in personalized medicine schemes. Moreover, it is possible to alter the properties of polymeric scaffolds for regenerative medicine to make them pH-responsive and tunable by the inclusion of our patchy nanoparticles.

### 3.3. Biocompatibility

**Figure 4:**
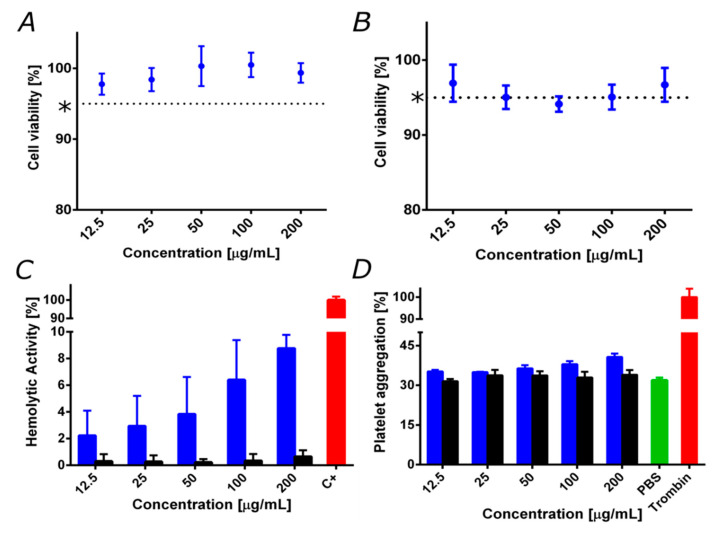
Biocompatibility assays for patchy magnetite/silver (blue) compared to bare magnetite (black). (*) magnetite cell viability reported elsewhere in the same 72 h and further [36,39]. (**A**) Cytotoxicity by lactate dehydrogenase enzyme (LDH) in Vero cells line after 24 h. (**B**) Cytotoxicity by LDH in Vero cells after 48 h. Cell viability remained above 95%. (**C**) The hemolysis assay shows an average hemolytic effect below 10% in all cases. The positive control was Triton 100-X. (**D**) Platelet aggregation assay shows an aggregation tendency of about 40%, which is similar to that of bare magnetite. The negative and positive controls were PBS 1X and thrombin, respectively.

To evaluate the viability of the developed patchy magnetite/silver nanoparticles as potential nanoplatforms for biological applications such as drug delivery to treat different pathologies, it is crucial to study potential biocompatibility issues carefully. To achieve this, cytotoxicity, hemolytic, and platelet aggregation tendencies were evaluated. Figure 4A and Figure 4B show the Vero cell viability after exposure to the magnetite/silver nanoparticles for 24 and 48 h, respectively. Patchy magnetite/silver nanoparticles showed high cell viability levels of about 95%, even after 48 h of incubation. Although silver is widely reported as a cytotoxic and genotoxic agent in mammalian cells [49], these results can be easily explained by the low silver content in the nanoparticles, as well as by the chemical interactions between the silver and the magnetite core that restrict the release of silver ions responsible for the reported cytotoxic activity [50]. Moreover, the obtained cell viability levels, compared to previously reported results of the cytocompatibility of magnetite nanoparticles (dotted line Figure 4A–B) [39], confirm that the silver shell shows no additional effect on the cytocompatibility.

In addition, Figure 4C shows the hemolytic activity of the magnetite and the magnetite/silver nanoparticles. It is possible to observe that magnetite nanoparticles presented negligible hemolysis, as evidenced by an average percentage below 1% for concentrations up to 200 μg/mL. In contrast, patchy magnetite/silver nanoparticles showed an average of 9% at the highest concentration (200 μg/mL). This result follows a typical dose–response tendency in which an increase in the concentration causes an increase in hemolytic activity. This agrees with previous studies that reported the high hemolytic effect for nanostructured silver [51].

Furthermore, Figure 4D shows the platelet aggregation tendency of the magnetite and the patchy magnetite/silver nanoparticles. Both types of nanoparticles presented a similar platelet aggregation percentage of about 30%. However, it is possible to observe that an increase in the concentration of patchy magnetite/silver nanoparticles leads to an increase in the platelet aggregation percentage. This is consistent with previous articles that establish a relationship between the amount of silver and the platelet aggregation tendency [52]. The obtained results confirm the high biocompatibility of the synthesized patchy magnetite/silver nanoparticles in terms of low hemolytic and platelet aggregation tendencies and low LDH cytotoxicity. The superior biocompatibility obtained after purification is a critical feature to fully enable some of the applications described above. This is particularly important for complying with the increasingly stringent medical device regulatory frameworks emerging worldwide [1,53].

## 4. Conclusions

Patchy bimetallic nanoparticles instead of monometallic ones allow more versatile and tunable surface chemistries, which could be attractive to enable numerous biological and biomedical applications. Issues regarding environmental impact and reduced biocompatibility are still to be overcome to realize their full potential at a larger scale. Here, we introduced a green synthesis method to prepare patchy silver/magnetite nanoparticles to overcome such issues. This was with the final intention of putting forward a versatile platform to enable multifunctional nanostructures with high biocompatibility levels. This was achieved by a highly efficient downstream purification process that allowed removing excess toxic reagents and the target nanostructures’ isolation. The success of each stage of the process was evaluated with the aid of UV-Vis, FTIR, and DLS. Successful conjugation of the pH-responsive polymer pDMAEMA on the patchy silver shell and the organosilane APTES on the magnetite core confirmed the suitability of the nanostructures for highly efficient conjugation. Finally, the nanostructures exhibit high biocompatibility in terms of hemolysis, platelet aggregation, and LDH cytotoxicity. In conclusion, our study also provides details for the conjugation of multiples chemistries on the surface of the patchy bimetallic nanoparticles, which might be useful for emerging applications in nanomedicine where high biocompatibility is of the utmost importance.
